# Two-Year Results of XEN Gel Stent Implantation for Pseudoexfoliative Glaucoma in Phakic versus Pseudophakic Eyes

**DOI:** 10.3390/jcm13144066

**Published:** 2024-07-11

**Authors:** Emil Nasyrov, David A. Merle, Caroline J. Gassel, Daniel A. Wenzel, Bogomil Voykov

**Affiliations:** Centre for Ophthalmology, University Hospital Tuebingen, Elfriede-Aulhorn-Str. 7, 72076 Tübingen, Germany

**Keywords:** pseudoexfoliative glaucoma, filtering surgery, minimally invasive surgery, XEN, phacoemulsification, minimally invasive bleb surgery, MIBS

## Abstract

**Objectives**: To investigate whether phakia affects the outcome of XEN-45 gel stent implantation in the treatment of pseudoexfoliative glaucoma (PXG). **Methods**: A retrospective, comparative cohort study of 30 phakic and 55 pseudophakic PXG patients who received the XEN-45 gel stent at a tertiary centre. The primary outcome measure was two-year success defined as a ≥20% lowering of intraocular pressure (IOP) and a target IOP of 6–21 mmHg. Success was complete without and qualified irrespective of antiglaucoma medication use. Further glaucoma surgery other than needling was regarded as a failure. The secondary outcome measures included changes in IOP, revision and complication rates. **Results**: The complete two-year success rates were 70% and 59% in the phakic and pseudophakic groups, respectively (*p* = 0.75, log-rank test), and the qualified rates were 80% and 72%, respectively (*p* = 0.89). The median IOP reduction from baseline was 54% in phakic, and 46% in pseudophakic eyes. While needling rates were similar, the incidence of early incisional bleb revisions was significantly higher in the phakic eyes (13% vs. 0% within 3 months; *p* = 0.0098, chi-square). Increasing after a year, significantly more pseudophakic eyes failed due to secondary glaucoma surgery (16% vs. 0%; *p* = 0.0191). **Conclusions**: The XEN-45 gel stent offers equally effective IOP control for both phakic and pseudophakic patients. However, the onset of bleb revisions and the necessity for secondary glaucoma surgery differed significantly between the groups.

## 1. Introduction

Progression in pseudoexfoliative glaucoma (PXG) is more rapid and aggressive, and response to medical therapy is poorer compared to primary open-angle glaucoma (POAG) [1]. PXG often requires earlier surgery in order to control intraocular pressure (IOP).

In PXG, the lens is thought to contribute to increased IOP and IOP fluctuations through iridolenticular friction, the release of pigment and exfoliation material, and anterior lens movement [2]. Accordingly, evidence demonstrates that phacoemulsification cataract extraction (PCE) can lower IOP more effectively in PXG patients compared to POAG patients after two years [3]. However, the IOP lowering effect of PCE diminishes in the long term [4]. On the other hand, PXG is a well-known risk factor for intraoperative and late postoperative complications after PCE such as lens and intraocular lens dislocation [5,6].

Trabeculectomy, regarded as the gold standard in glaucoma surgery, results in higher long-term failure rates and poorer IOP control in PXG patients compared to POAG patients [7,8,9]. The subconjunctival drainage of pseudoexfoliation material and other pro-inflammatory factors is associated with more pronounced bleb scarring in PXG eyes [2,7]. The influence of the lens status on trabeculectomy outcomes is controversial. Several studies involving cohorts of mixed glaucoma types, including those with variable proportions of PXG patients, found that pseudophakia was a risk factor for failure [10,11]. However, other studies found no influence of previous cataract surgery on the success of trabeculectomy [12,13]. Interestingly, one study reported phakia in PXG patients as a risk factor for failure [14].

Microinvasive bleb-forming surgery (MIBS) with the XEN-45 gel stent (Allergan; AbbVie Inc., North Chicago, IL, USA) was developed to reduce complications associated with traditional filtering surgery and has been demonstrated to result in comparable success rates [15,16]. Contrary to trabeculectomy, one prospective study found that outcomes for XEN implantation were similar in PXG and POAG eyes [17]. The authors hypothesise that these findings might be explained by a greater restriction of pro-inflammatory factors reaching the subconjunctival space due to the defined stent lumen, the less invasive ab interno approach, and the removal of pseudoexfoliation material. However, most PXG eyes in that study received combined XEN and PCE (n = 40), and 11 out of 13 eyes that received a standalone XEN implantation had previously undergone cataract surgery [17]. Thus, no subgroup analysis of phakic patients without simultaneous or prior cataract surgery was possible in that study. Interestingly, the combined PCE and XEN-45 gel stent implantation did not influence the outcome for patients with PXG [17,18]. 

A limited number of studies have investigated the influence of previous PCE on long-term surgical success and bleb function following MIBS. A prospective multi-centre trial by Fea et al. reported a more pronounced decrease in IOP and a higher percentage of patients with lower IOP levels after standalone XEN implantation (both with and without previous PCE) compared to those who had undergone a combined PCE-XEN procedure after one year [19]. Additionally, significantly higher rates of needlings were observed after combined PCE-XEN procedures compared to standalone XEN implantations, and in pseudophakic eyes compared to phakic eyes in a standalone subgroup analysis. However, this study involves a cohort of mixed glaucoma types with the majority being POAG patients. Thus, it remains unknown whether XEN-45 implantation is effective specifically in patients with PXG without previous PCE. Therefore, this study aims to evaluate the success and safety of XEN-45 and the role of the preoperative lens status in PXG eyes in a real-world setting.

## 2. Materials and Methods

This was a retrospective, comparative cohort study of phakic and pseudophakic PXG patients who underwent XEN-45 gel stent implantation at a single tertiary centre between 2016 and 2021. Surgery was performed in patients considered as having above-target IOP despite maximum tolerated medical therapy. This study was conducted in accordance with the tenets of the Declaration of Helsinki. Ethical approval was granted by the University of Tuebingen’s local institutional ethics committee (project number: 747/2022BO2; approval date: 23 November 2022). Due to the retrospective design, the ethics committee waived the requirement for patient consent to use the data in this study. 

Inclusion and exclusion criteria: All consecutive patients diagnosed with PXG who underwent XEN implantation were eligible for inclusion. Patients who had previously undergone filtering glaucoma surgery and/or received combined PCE and XEN implantation were excluded. Those with a postoperative follow-up period of fewer than 12 months were excluded. Regarding phakic patients, eyes that received PCE within the first year after XEN and thus had a follow-up of fewer than 12 months were excluded. As a tertiary referral centre, patients are routinely followed postoperatively after 1, 3, 6, and 12 months and then annually. Additional follow-up visits can be scheduled by the referral doctor. Patients are usually re-referred in cases with suspected disease progression or loss of IOP control. 

Surgical technique: Surgery was performed by a single experienced surgeon under topical anaesthesia using oxybuprocaine eye drops, as described previously [20]. Briefly, a small bubble of 5 µg mitomycin C (MMC) (seven patients received 10 µg, and three patients received 20 µg) was injected into the subconjunctival space superonasally. A clear cornea incision was then created 1 mm inferotemporally, and a smaller side-port incision was made 3 clock-hours from the first incision. The anterior chamber was then filled with viscoelastic (HEALON^®^, Abbott Laboratories Inc., Abbott Park, IL, USA). Following this, the XEN-45 injector was inserted through the main incision into the anterior chamber, and the needle penetrated through the sclera into the subconjunctival space, 3 mm from the limbus. The stent was then released, and the injector was withdrawn. The viscoelastic was then washed out, resulting in the formation of a filtering bleb. Finally, cefuroxime was injected into the anterior chamber. 

Pre- and postoperative management: The baseline IOP was assessed under antiglaucoma medication. The medication was stopped two weeks before surgery, and oral acetazolamide (500 mg) was started two to three times daily and stopped on the day of surgery. Unpreserved dexamethasone eye drops were administered three times daily one week before surgery. On the first postoperative day, moxifloxacin eye drops were administered four times daily for two weeks, and unpreserved dexamethasone eye drops were administered five times daily starting from postoperative day one, tapering down over 6–8 weeks. If post-surgical IOP levels were considered inadequate and clinical signs of bleb scarring were detected, a needling procedure was attempted first instead of re-initiating antiglaucoma medication. Needling was performed in the operating theatre under topical anaesthesia using oxybuprocaine eye drops, as described previously [20]. An incisional bleb revision was performed if the XEN stent was not visible due to severe bleb fibrosis.

Study measures: Follow-up visits occurred on the first two postoperative days, after one, three, and six months, and then annually. These visits included a complete ophthalmological examination that included IOP measurements using Goldmann applanation tonometry as well as slit lamp and fundus examinations. Perimetry was performed using the Octopus 900 perimeter (Haag-Streit, Koeniz, Switzerland). 

The primary outcome measure was surgical success after two years. Success was defined as ≥20% IOP reduction from baseline within the range of 6–21 mmHg (Category A), which aligns with the guidelines set by the World Glaucoma Association [21]. Additional upper IOP cut-off points were set at IOP ≤ 18 mmHg (Category B) and ≤15 mmHg (Category C). If patients did not meet the success criteria at two consecutive visits starting from the first month, failure was recorded on the first visit in which the criteria were not met. Furthermore, success was considered complete if it was achieved without antiglaucoma medication, and success was considered qualified if the criteria were met regardless of whether or not topical IOP-lowering medication was used. Loss of light perception and/or the need for incisional bleb revision or further glaucoma surgery were considered failures. Needling procedures were not regarded as failures [21]. Phakic patients who received PCE were censored at the time of surgery in regard to survival analysis.

Secondary outcome measures included the reduction of median IOP compared to baseline, the number of individual antiglaucoma agents used, the rates of interventions (needlings, incisional bleb revisions, and further glaucoma surgery), and the complication rates. Patients of both groups who received secondary glaucoma surgery, and phakic patients who received PCE were censored at the time of secondary surgery/PCE. Further secondary measures collected after censoring were excluded from the overall analysis.

Statistical analysis: Surgical success was evaluated using Kaplan–Meier survival estimates and compared using the log-rank test. For time-to-event analysis of bleb revisions, the Gehan–Breslow–Wilcoxon test was used with significantly different revision rates between groups at earlier and later time points indicating a violation of the proportional hazards assumption. For the analysis of secondary glaucoma surgery, the log-rank test was used. The D’Agostino and Pearson omnibus normality test was used to test for the normal distribution of data, which indicated that IOP and the number of medications used were not normally distributed. Accordingly, postoperative changes within groups were analysed using the Wilcoxon matched-pairs signed rank test, while the differences between the groups were analysed using the Mann–Whitney test. For normally distributed data, paired and unpaired *t*-tests were used. The categorical data were compared using the chi-square test. A probability value of *p* < 0.05 was considered statistically significant. 

## 3. Results

### 3.1. Study Participants

One hundred and four eyes with PXG which underwent XEN implantation were eligible for inclusion. Seven eyes were excluded due to previous incisional glaucoma surgery and eleven others due to combined PCE and XEN implantation. One patient received PCE 4 months after XEN surgery and was excluded. In total, 55 pseudophakic and 30 phakic eyes from 47 and 27 patients, respectively, were included in the final analysis. The mean follow-up time ± standard deviation was 23 ± 13 months in the phakic vs. 25 ± 12 months in the pseudophakic group. Four eyes in the phakic group received PCE after a median of 14.5 months (range 13–16) and were censored at the time of surgery. Table 1 summarises the patients’ demographic and clinical characteristics. The pseudophakic patients were significantly older. However, other baseline characteristics, especially preoperative median IOP, number of medications and mean MD, were similar in both groups. 

### 3.2. Effectiveness

**Figure 1:**
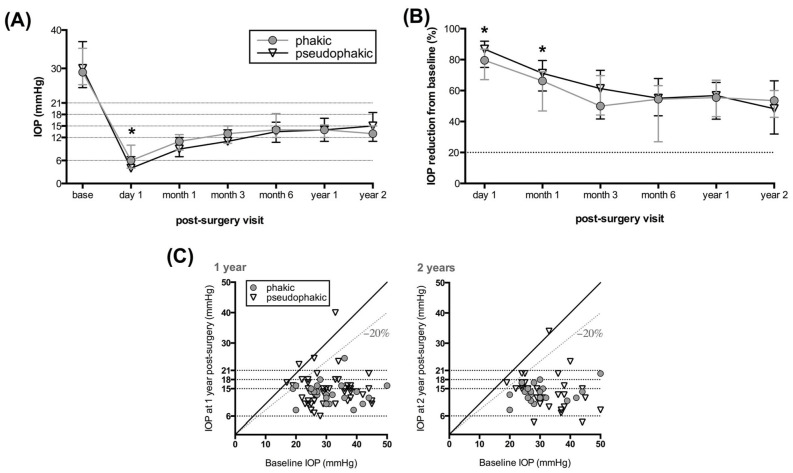
Changes in postoperative intraocular pressure (IOP). The median IOP with the interquartile range (IQR; (**A**)) and median IOP reduction from baseline with IQR (**B**) are plotted across post-surgery visits between the phakic and pseudophakic eyes. The dotted horizontal lines demarcate the three IOP target ranges and the IOP reduction target in (**A**,**B**), respectively. Individual IOP readings at one and two years (**C**) are compared against the medicated baseline values. The diagonal line implies no change, the dotted diagonal line demarcates a 20% IOP reduction, and the horizontal lines indicate post-surgery IOP targets. Differences between groups were tested using the Mann–Whitney test. Asterisks indicate *p* < 0.05.

The median IOP in both the phakic and the pseudophakic eyes was significantly lower after XEN implantation on all post-surgery visits (*p* < 0.0001 for each visit, Wilcoxon matched-pairs signed rank test; Figure 1A). In the phakic group, the median IOP (interquartile range [IQR]) was lowered from 29 mmHg (26–35 mmHg, n = 30) at baseline to 14 mmHg after one year (12–15 mmHg, n = 26) and to 13 mmHg after two years (12–15 mmHg, n = 20). In the pseudophakic group, the median IOP (IQR) decreased from 30 mmHg (26–37 mmHg, n = 55) to 14 mmHg (11–17 mmHg, n = 49) after one year and to 15 mmHg (12–19 mmHg, n = 28) after two years. The median IOP reduction from baseline (IQR) was 56% (43–67) and 54% (43–60) in the phakic group and 57% (42–65) and 46% (32–66) in the pseudophakic group one and two years post-surgery, respectively (Figure 1B). At the first day visit, the pseudophakic eyes had a significantly lower median IOP (4 mmHg, IQR 3–7 vs. 6 mmHg, IQR 4–10; *p* = 0.0469, Mann–Whitney test) and higher median IOP reduction from the baseline than the phakic eyes (87%, IQR 75–92 vs. 80%, IQR 67–87; *p* = 0.0494; Figure 1A,B). At 1 month, the median IOP reduction was significantly higher in the pseudophakic group (71%, IQR 60–79 vs. 66%, IQR 47–72; *p* = 0.0428; Figure 1B). No other differences in the median IOP or IOP reduction were found between the two groups. Figure 1C depicts individual IOP readings during the one- and two-year follow-up visits compared to baseline values.

The median number of antiglaucoma medications used was significantly lower in both groups in all post-surgery visits (each *p* < 0.0001, Wilcoxon matched-pairs signed rank test). The median number of medications (IQR) was significantly reduced in the phakic eyes from 3 (3–4) at baseline to 0 (0–0) after one year and two years, and this was reduced from 4 (3–4) to 0 (0–1) after one year and two years in the pseudophakic eyes. The Mann–Whitney test indicated a significantly higher median number of medications in the pseudophakic group at one year (*p* = 0.0474) and two years (*p* = 0.0276) but not on previous follow-up visits. In the phakic group, 85% and 95% were medication-free, while in the pseudophakic group, 71% and 70% were medication-free after one and two years, respectively. No patient required systemic carbonic anhydrase inhibitors during the follow-up period.

**Figure 2:**
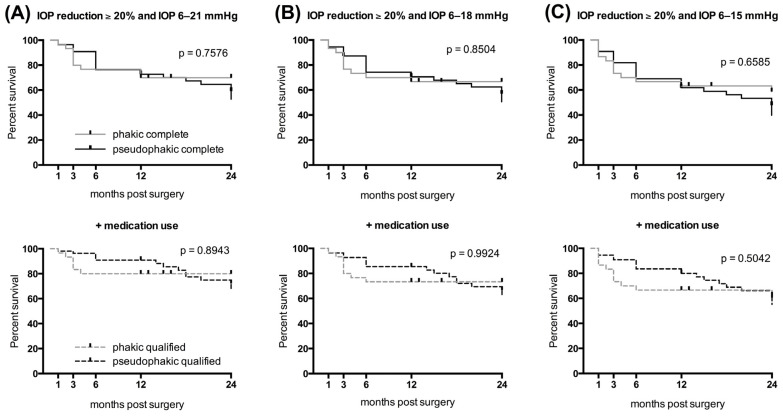
The Kaplan–Meier survival probabilities for surgical success in the phakic and pseudophakic eyes. Surgical success using the three IOP target ranges of (**A**) 6–21 mmHg, (**B**) 6–18 mmHg, and (**C**) 6–15 mmHg. Success was considered complete without the use of additional antiglaucoma medication and qualified (the dotted lines) irrespective of the use of additional antiglaucoma medication. Differences between groups were tested using the log-rank test.

Complete surgical success with an IOP target range between 6–21 mmHg was achieved in 70% of the phakic eyes after both one and two years and in 73% and 59% of the pseudophakic eyes, respectively (Figure 2A). Qualified success was achieved in 80% of the phakic group after one and two years and in 91% and 72% of the pseudophakic group, respectively (Figure 2A). 

Regarding the postoperative IOP target of 6–18 mmHg, the complete success rates were 67% after one and two years in the phakic group, and 71% and 60% in the pseudophakic group, respectively (Figure 2B). The qualified success rates after one and two years were both 73% in the phakic group and 85% and 67%, respectively, in the pseudophakic group (Figure 2B). 

The lowest IOP target of 6–15 mmHg was achieved without medication use in 63% and 62% of the phakic and pseudophakic eyes after one year and 59% and 48% after two years, respectively (Figure 2C). The qualified success rates for the phakic eyes were 67% and 62% after one and two years and 80% and 61% for the pseudophakic eyes, respectively (Figure 2C). No statistical differences were found between the groups across any success category using the log-rank test (Figure 2). 

### 3.3. Postoperative Complications and Interventions

The complication and intervention rates are summarised in Table 2. The requirement for viscoelastic injection due to hypotony was numerically but not statistically more common in the pseudophakic eyes (Table 2). Intraocular lens (IOL) dislocation was observed in one patient, and uveitis-glaucoma-hyphaema (UGH) syndrome occurred in three (7%) pseudophakic patients of whom two required IOL removal. Blebitis with endophthalmitis and subsequent loss of light perceptions were observed in one pseudophakic patient due to untreated bacterial conjunctivitis and late referral to our clinic. Furthermore, malignant glaucoma and retinal detachment were each observed once. Four phakic patients (13%) developed significant cataracts (defined as loss of BCVA ≥ 2 lines) and received PCE after a median of 14.5 months (range 13–16) after XEN implantation. 

### 3.4. Postoperative Bleb Interventions and the Need for Additional Surgery

**Figure 3:**
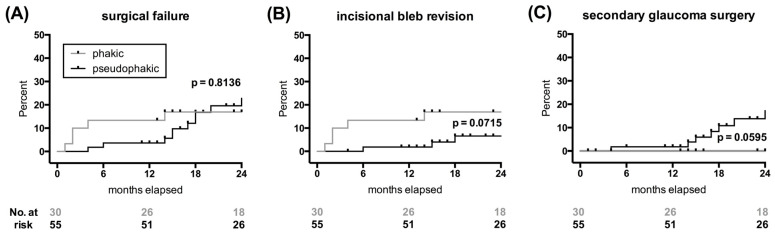
The surgical failures due to incisional bleb revision and additional glaucoma surgery. The Kaplan–Meier estimates were used for time-to-event analyses of surgical failures (**A**), which included incisional bleb revision (**B**) and additional glaucoma surgery (**C**). The tick marks indicate censored patients either due to the end of follow-up (**A**–**C**) or due to the requirement for glaucoma surgery in (**B**) and incisional bleb revision in (**C**). Differences between groups were tested using the log-rank test (**A**,**C**), and the Gehan test (**B**).

Table 3 shows rates and onset of bleb interventions and additional glaucoma surgery for both groups. The proportion of eyes that did not require any bleb intervention and proportions for individual bleb interventions during the comprehensive follow-up period were similar. Nonetheless, the rate of incisional bleb revisions performed within the first three months after surgery was significantly higher in the phakic group compared to the pseudophakic group (13% vs. 0% of, *p* = 0.0055, chi-square test). Time-to-event analysis indicated a trend between groups (*p* = 0.0715, Gehan–Breslow–Wilcoxon test; Figure 3A). On the other hand, significantly more pseudophakic eyes had a necessity for additional glaucoma surgery (16% vs. 0% of, *p* = 0.0191, chi-square test), which increased one year after XEN implantation (*p* = 0.0595, log-rank test; Figure 3B). For overall surgical failure, which combined failures due to both incisional bleb revision and additional glaucoma surgery, no statistical difference was found between the phakic and pseudophakic eyes (*p* = 0.8136; Figure 3C). Lastly, the timing of needling procedures was similar between groups (Table 3).

## 4. Discussion

This study aims to investigate the effectiveness and safety of XEN implantation in PXG patients without previous PCE, which has not been reported yet. Our results demonstrate that XEN-45 gel stent implantation is equally effective for IOP control in phakic and pseudophakic PXG patients. Both groups achieved comparable success rates across all categories. Moreover, the rates for the pseudophakic patients are similar to the published results that employed the same success criteria on standalone XEN implantation in PXG patients [17]. Additionally, the success rates are comparable to results for trabeculectomy with MMC in PXG patients [7,8]. 

Two years after XEN implantation, surgical failure rates were similar in both phakic and pseudophakic PXG eyes in our study. However, the chronological sequence was different between the groups. Phakic eyes that failed did so within six months after surgery due to distinct bleb fibrosis, and pseudophakic eyes due to secondary glaucoma surgery increased in the second year, while rates and timing of needling interventions were comparable between the groups. 

Our results indicate phakia might be a risk factor for early bleb fibrosis. Interestingly, this is in contrast to the higher bleb revisions in pseudophakic patients indicated by Fea et al., who found a statistically significant but only marginally higher needling rate in pseudophakic compared to phakic eyes after standalone XEN implantation (45.83% vs. 44.19%) in a cohort of mostly POAG patients [19]. However, our results align with those of Tan et al., who found a higher number of phakic patients requiring bleb interventions, and Widder et al., who found that more pseudophakic than phakic eyes achieved IOP targets without the need for further incisional bleb surgery, both studies in mixed cohorts [22,23]. However, comparing our success or revision rates with those of Widder et al. is not feasible, as their study design did not allow for additional glaucoma medication use, and incisional bleb surgery was indicated to achieve IOP targets and was not regarded as a failure. In contrast, we defined incisional bleb revision as a failure, performing it only when the XEN stent was not visible under the conjunctiva at the slit lamp due to fibrosis. 

In our study, the pronounced scarring effect in phakic patients requiring higher rates of incisional bleb revisions led to a steeper decline in the success rates in the phakic patients compared to the pseudophakic patients. After the first six months, however, success rates in the phakic group remained stable across all categories. These findings indicate that the higher risk for bleb fibrosis after XEN implantation might be temporally restricted and that phakic patients with PXG should be more closely observed in the early postoperative phase.

The role of the lens status for trabeculectomy is controversial, and significant age differences between groups confound studies [10,11,12,13,14,24]. Phakia has been reported to be a risk for trabeculectomy failure in patients with PXG [14]. However, studies that included mixed types of glaucoma have reported either no influence of the lens status on the efficacy of trabeculectomy [13], or pseudophakia as a risk factor for long-term failure [10]. The latter finding has been discussed as potentially caused by the increased pro-inflammatory cytokines such as monocyte chemoattractant protein-1 (MCP-1) in POAG and interleukin-8 and MCP-1 in PXG, which were found to be elevated one year following PCE [24]. We found that failure rates in pseudophakic compared to phakic eyes increased one year after XEN implantation, which could be attributable to the continuously altered aqueous humour microenvironment in pseudophakic PXG eyes as reported by Inoue et al. [24]. Our failure rate of 16% due to secondary glaucoma surgery in pseudophakic eyes is comparable to the published 2-year results of XEN implantation in PXG patients [17].

Several studies in patients with mixed glaucoma types have demonstrated that combined XEN-PCE was equally or less effective than standalone XEN implantation [19,22,25]. For PXG specifically, no differences in surgical outcomes have yet been demonstrated [17,18], but a two-stage surgical approach might still be favourable for PXG due to longer surgery times and generally higher complication rates attributable to combined procedures [26]. In this regard, whether XEN implantation should be performed before or after PCE has yet to be established. This real-world study reflects and is confounded by the age difference found among phakic and pseudophakic patients and cannot finally answer this question. Our results suggest that performing cataract surgery first might minimise the risk of bleb failure. However, conducting lens extraction first also increases the lifetime risk of late complications, such as in-bag IOL dislocation [6,26]. In the current study, we observed one case of spontaneous IOL dislocation and three cases of UGH syndrome that required IOL removal in two of the pseudophakic patients. These complications and the surgical approaches for their treatment pose considerable risks for the long-term bleb function in pseudophakic patients. 

In PXG, cataract extraction has been demonstrated to effectively lower IOP and has recently been investigated as a feasible first treatment option instead of antiglaucoma medication use [3,27]. In regard to whether prior cataract surgery might have an additive effect on XEN implantation, we only found a minor IOP-lowering effect in pseudophakic compared to phakic eyes, limited to the first month after surgery in our study. However, higher rates of early bleb fibrosis and subsequent temporary loss of IOP control observed in phakic patients might be associated with this finding and could have exaggerated a minor IOP-lowering effect in pseudophakic patients.

The main limitation of our study is that age significantly differs between the groups. This is attributable to age being an inherent confounder of previous cataract surgery in real-world settings. Significant age differences are also found among all similar studies comparing phakic and pseudophakic patients in the context of filtering surgery [10,11,12,13,14,24]. Preoperative age is widely regarded as a predictive factor for failure after trabeculectomy [28,29]. However, previous studies have not found age to be a predicative factor for failure or for higher rates of needling interventions after XEN implantation [18,30]. Additionally, the PXG phenotype rapidly evolves with age [31]. However, despite the age difference in the present study, both groups matched glaucoma characteristics such as baseline IOP, number of medications and MD. We refrained from conducting active group matching for age since groups were matching in regard to glaucoma characteristics, and matching would have introduced selection bias towards older pseudophakic and younger phakic patients. It was not the aim to investigate the optimal timing for PCE in MIBS, which would require a controlled study design. Second, most patients were treated with 5 µg MMC. However, some received 10 µg (n = 7) or 20 µg (n = 3). The low number of patients, who received higher doses, did not allow for a sub-analysis. However, our data (submitted) revealed no dose-dependent difference in the long-term success rates for patients with open-angle glaucoma. 

## 5. Conclusions

This real-world study included two cohorts of patients with similar glaucoma manifestations who had not undergone prior filtering glaucoma surgery. To our knowledge, this is the first study comparing the efficacy and safety of XEN gel stent implantation in phakic and pseudophakic PXG eyes. Our results demonstrated that ab interno XEN-45 gel stent implantation was equally effective for IOP control in phakic and pseudophakic eyes. However, risks for incisional bleb interventions and additional glaucoma surgery, as well as their timing, differed between the groups and should be taken into account during follow-up. 

In the context of emerging personalised healthcare, our findings can play an important role in the management of these patients and might aid the design and sample size calculations of future controlled studies investigating the timing of cataract surgery in MIBS.

## Figures and Tables

**Table 1:** Patients’ demographic and clinical characteristics and previous surgical history.

Characteristic	Phakic (n = 30)	Pseudophakic (n = 55)	*p*
Median age in years (IQR)	67 (60–71)	77 (73–81)	<0.0001 ^1^
Female sex (%)	n = 14 (47%)	n = 34 (62%)	0.1782 ^2^
White ethnicity (%)	n = 30 (100%)	n = 55 (100%)	
Median preoperative medicated IOP in mmHg (IQR)	29 (26–35)	30 (25–37)	0.6752 ^1^
Median number of preoperative medications (IQR)	3 (3–4)	4 (3–4)	0.4170 ^1^
Mean preoperative visual field ± SD (MD in dB)	−9 ± 5.6	−10 ± 5.6	0.4651 ^3^
MD ≥ −6.0 dB (% of eyes)	26%	31%	
MD < −6.0 dB and ≥−12.0 dB (% of eyes)	38%	38%	
MD < −12.0 dB (% of eyes)	36%	31%	
Ophthalmological comorbidity			
Epiretinal membrane	n = 2 (7%)	n = 2 (4%)	0.5284 ^2^
Secondary IOL implantation	Not applicable	n = 2 (4%)	
Previous glaucoma interventions			
None	n = 18 (60%)	n = 33 (60%)	1.0
SLT/ALT	n = 8 (27%)	n = 13 (24%)	0.7569 ^2^
Laser peripheral iridotomy	n = 0	n = 1 (2%)	0.4575 ^2^
CPC	n = 0	n = 3 (5%)	0.1928 ^2^
Canaloplasty	n = 4 (13%)	n = 4 (7%)	0.3605 ^2^

The number of medications used was calculated based on individual active agents. ALT = argon laser trabeculoplasty; CPC = cyclophotocoagulation; dB = decibel; IQR = interquartile range; MD = mean defect; SD = standard deviation; SLT = selective laser trabeculoplasty. Statistical tests indicated by: ^1^ the Mann–Whitney test, ^2^ chi-square test, and ^3^ unpaired *t*-test.

**Table 2:** Postoperative complications and interventions.

Observed Complication	Phakic (n = 30)	Pseudophakic (n = 55)	*p*
Postoperative hyphaema	n = 7 (23%)	n = 14 (25%)	0.8258
Injection of viscoelastic	n = 3 (10%)	n = 10 (18%); (n = 5 twice)	0.3166
Early hypotony (<1 month post-surgery)	n = 0	n = 2 (4%)	0.2905
Sustained hypotony (>3 months post-surgery)	n = 0	n = 0	
Stent occlusion (spontaneous resolution)	n = 1 (3%)	n = 1 (2%)	0.6596
Conjunctiva dehiscence requiring suturing	n = 1 (3%)	n = 0	0.1732
Iris incarceration in the stent	n = 0	n = 1 (2%)	0.4575
Malignant glaucoma	n = 0	n = 1 (2%)	0.4575
Rhegmatogenous retinal detachment	n = 1 (3%)	n = 0	0.1732
Spontaneous IOL dislocation	Not applicable	n = 1 (2%)	
UGH syndrome	Not applicable	n = 3 (5%)	
Subsequent IOL removal		n = 2 (4%)	
Cataract with BCVA loss ≥2 lines	n = 4 (13%)	Not Applicable	
Cataract surgery	n = 4 (13%)	Not applicable	
Late bleb leak	n = 0	n = 0	
Blebitis, endophthalmitis	n = 0	n = 1 (2%)	0.4575
Loss of light perception (following blebitis)	n = 0	n = 1 (2%)	0.4575

IOL, intraocular lens; UGH, uveitis-glaucoma-hyphaema syndrome; BCVA = best corrected visual acuity; the differences between the groups in terms of the complication and intervention rates were analysed using a chi-square test.

**Table 3:** Rates of bleb interventions and additional glaucoma surgery.

	Phakic (n = 30)	Pseudophakic (n = 55)	*p*
No bleb intervention	n = 13 (43%)	n = 24 (44%)	0.9785
Number of needlings with MMC per eye:			
≥1	n = 17 (57%)	n = 31 (56%)	0.9785
1	n = 7 (23%)	n = 16 (29%)	0.5680
2	n = 6 (20%)	n = 12 (22%)	0.8446
3	n = 2 (7%)	n = 2 (4%)	0.5284
4	n = 2 (7%)	n = 1 (2%)	0.2470
Number of incisional bleb revisions per eye:			
0	n = 25 (87%)	n = 51 (93%)	0.1786
1	n = 4 (13%)	n = 4 (7%)	0.1786
2	n = 1 (3%)	n = 0	0.1732
Additional glaucoma surgery			
No additional glaucoma surgery	n = 30 (100%)	n = 46 (84%)	0.0191
XEN-45 implantation	n = 0	n = 4 (7%)	0.1392
PreserFlo MicroShunt	n = 0	n = 2 (4%)	0.2905
Trabeculectomy	n = 0	n = 3 (5%)	0.1928
Other surgery			
Cataract surgery	n = 4 (13%)	Not applicable	
IOL removal	n = 0	n = 2 (4%)	
Vitrectomy	n = 0	n = 1 (2%)	
Total number of needlings (individual eyes)	n = 33 (17 eyes)	n = 49 (31 eyes)	
<3 months postop.	n = 8 (8)	n = 14 (12)	0.6145
3–6 months postop	n = 10 (6)	n = 8 (8)	0.5170
6–12 months postop	n = 6 (4)	n = 7 (6)	0.7403
12–24 months postop.	n = 2 (2)	n = 13 (12)	0.0719
>24 months postop.	n = 7(4)	n = 7(6)	0.7403
Total number of incisional bleb revisions (individual eyes)	n = 6 (5 eyes)	n = 4 (4 eyes)	
<3 months postop.	n = 5 (4)	n = 0	0.0055
3–6 months postop	n = 1	n = 1	0.6596
6–12 months postop	n = 0	n = 0	
12–24 months postop.	n = 0	n = 2 (2)	0.2905
>24 months postop.	n = 0	n = 1	0.4575

MMC = mitomycin c; the differences between the groups’ intervention rates were analysed using a chi-square test.

## Data Availability

The data presented in this study are available upon reasonable request from the corresponding author.
